# Crystal structure analysis of anti-V2 mAb 2158 suggests a conformational epitope involving an N-linked glycan

**DOI:** 10.1186/1742-4690-9-S2-P70

**Published:** 2012-09-13

**Authors:** B Spurrier, R Pan, J Sampson, C Williams, M Gorny, S Zolla-Pazner, X Kong

**Affiliations:** 1 NYU School of Medicine, New York, NY, USA; 2 Veterans Affairs New York Harbor Healthcare System, New York, NY, USA

## Background

Many structural elements of HIV-1 gp120 have been revealed by X-ray crystallography, NMR and electron microscopy; however, several key immunogenic regions are still not well understood. Data from the recent RV144 trial indicated that antibodies targeting the V1V2 region correlate with a lower risk of infection. A detailed characterization of anti-V2 antibodies, in concert with recent V1V2 structural information is critical to the design of a V2 immunogen.

## Methods

We have determined a crystal structure of the uncomplexed Fab fragment of anti-V2 mAb 2158. To understand the antigen binding mode of mAb 2158, we computationally created twenty V1V2 models from a panel of gp120s that were tested previously in ELISA for binding reactivity with mAb 2158. Subsequently, we docked these models in silico to the antigen binding site of mAb 2158.

## Results

The structure of Fab 2158 revealed that its antigen binding site consists of a hydrophobic surface comprised with residues from CDRs H2 and H3 with a deep pocket between CDRs H1 and H3. Surface energy analysis suggests that the heavy chain and the binding pocket are likely to dictate the antigen binding mode of Fab 2158. Our model-building of the 20 V1V2 structures revealed that the residues previously suggested as the mAb 2158 epitope all mapped to a single face on the V2 domain next to glycosylation site N186. Correlating our computational analysis with ELISA data suggests that the glycosylation of N186 plays a key role in binding and our docking results indicates that the mAb 2158 binding pocket can accommodate the mannose glycan harbored by N186.

## Conclusion

Structural analyses of Fab 2158 suggest that binding to a conformational epitope containing an N-linked glycan. Our results allow us to hypothesize a binding motif signature for anti-V2 mAb 2158 and form a framework for designing V2 immunogens.

